# Letter to the editor regarding an autosomal recessive mutation in SCL24A4 causing enamel hypoplasia in Samoyed and its relationship to breed-wide genetic diversity

**DOI:** 10.1186/s40575-018-0059-7

**Published:** 2018-05-01

**Authors:** Frank W. Nicholas, Cathryn Mellersh, Tom Lewis

**Affiliations:** 1 Emeritus Professor of Animal Genetics, Sydney School of Veterinary Science, University of Sydney, Sydney, NSW 2006 Australia; 2 Head of Canine Genetics, Animal Health Trust, Lanwades Park, Newmarket, CB8 7UU UK; 3 Genetics Research Manager, The Kennel Club, Clarges Street, London, W1J 8AB UK

To the editors,

We are writing in relation to the article ‘An autosomal recessive mutation in SCL24A4 causing enamel hypoplasia in Samoyed and its relationship to breed-wide genetic diversity’ published in Canine Genetics and Epidemiology on 22nd November 2017. Our chief concerns relate to the methodology, namely that: 1) using a small number (33) of microsatellite markers that cover only 66% (25/38) of autosomes is suboptimal for determining genetic variation occurring across the genome; and more seriously 2) the sample of dogs from which “breed-wide genetic diversity” was estimated contained a substantial but undisclosed number of close relatives (and so was not a random sample nor representative of the wider breed), meaning that findings pertaining to genetic variation cannot safely be extrapolated without qualification.

The study used microsatellite markers to estimate the genetic diversity of the Samoyed breed. While microsatellites have previously been used to this end, they have largely been superseded by dense SNP chips, where the quantity of bi-allelic marker data provides more precise information on the extent of genetic variation via homozygosity. The study used 33 short tandem repeats (STRs) on 25 autosomes, which is an average of 1.32 STRs per autosome included. However, since *Canis familiaris* has 38 autosomes, there was no means in this study to determine genetic variation across 1/3 of the genome. It is highly likely that there is genetic variation present within the Samoyed breed on these 13 autosomes, but this study is simply unable to detect its extent. Furthermore, genetic variation at genomic regions on the 25 included autosomes not in linkage disequilibrium with the STRs also cannot be detected. Thus, while this number of STRs may be sufficient for their stated purpose (parentage verification and forensic testing) and an acceptable ‘broad brush’ means of estimating diversity in a population where there is no alternative (such as wild populations), they are unable adequately to describe the extent of genetic variation across the canine genome, including regions of depleted variation due to selection. Also, it is not immediately evident how the cited website (endnote 14) justifies the claim that “this population [of 182 animals] would identify over 95% of existing genetic diversity and heterogeneity in Samoyed based on experience with other breeds”.

There is also an important issue regarding sampling of the 182 animals in relation to the inference of genetic variation at the 33 STRs in the wider, global breed. Little detail is given regarding the sampling and the likely bias therein. The authors state that “Samples were solicited through web communications and owners/breeders wishing to submit DNA for testing” and report that the sample comprised 144 dogs from North America, 32 from Europe and 6 from Australia (79.1%, 17.6% and 3.3%, respectively). The preponderance of North American dogs in the sample could imply the sample largely consisted of a (partially inter-related) subsection of the wider breed. Furthermore, given the method of sampling it is conceivable, or likely even, that repeat customers may have resulted in several individual breeders submitting samples from multiple, closely related dogs in their kennels or lines. Indeed, the authors state in the results section that “twenty of 168 (12%) healthy dogs were found to be heterologous [sic] for the mutation and most were parents or known close relatives of affected dogs”, thereby revealing that a significant section of the sample did indeed consist of close relatives, which would have an impact on detected genetic variation. However, other than reporting in the abstract that “This population was biased towards close relatives” as a caveat when estimating the population-wide incidence of enamel hypoplasia, the authors provide no further information, such as the degree of relationship among dogs within the unaffected or affected cohorts, which were nevertheless used to determine breed-wide genetic diversity. The authors report that they sought pedigree data and information on relationship to affected dogs, yet provide no evidence of the degree of relationship, as determined by pedigree, of this sample. This is critical to interpretation of the results; the interpretation would be very different if all 182 dogs were part of a large extended family compared to if they were unrelated [as determined from pedigree records] at 5 generations. The authors may point to their internal relatedness (IR) results, with one quarter of Samoyed IR scores between 0.132 and 0.502 putatively indicating a significant degree of parental relatedness, but there is no way of knowing the extent to which this level of relatedness is due to the sampling, as distinct from a reflection of the whole breed. In the conclusion, the authors state that “Samoyed have a lower level of genetic diversity than estimated from prior pedigree or SNP-based studies”, but this finding is to be expected given the inability to detect genome-wide variation in the methods employed and a sample that, by the authors’ own admission, contains a number of closely-related dogs.

The authors advise in the paper that “the mutation [for SCL24A4] could be safely eliminated without affecting existing genetic diversity”. Leaving aside the question of whether this paper provides a realistic estimate of genetic diversity, we would caution against the wholesale exclusion of carriers for breeding for two reasons. Firstly, with the availability of a DNA test for the mutation, breeders have the means to ensure that no affected puppy will ever be produced. So long as the mutation is not under indirect positive selection, the mutation frequency in this scenario is expected to remain static (apart from the chance of new versions of the same variant arising by mutation, which is exceedingly low). Therefore, it is a sensible course of action to make use of the available DNA tests to retain heterozygotes (carriers) in the breeding pool, at least for the first few generations after the DNA test becomes available, thereby decreasing the possibility of an artificially created genetic bottleneck which may lead to a rapid loss of genetic diversity. Selection against the mutation, by using carriers in a decreasing proportion of matings over the course of a number of generations, may still be applied and would result in a gradual reduction in the mutation frequency. Secondly, there are a number of independent selection objectives (even considering only those pertinent to health) in the Samoyed and most other breeds. There are several DNA tests commercially available to Samoyed breeders, including for degenerative myelopathy, hereditary nephritis, X-linked progressive retinal atrophy and oculo-skeletal dysplasia, as well as recommendations to participate in hip dysplasia screening. Because these objectives are most likely independent in genetic aetiology, identifying suitable breeding animals by excluding all those known to be carriers of any of the identified mutations influencing these disorders and that fall below a particular threshold for hip dysplasia would result in a cumulative effect, leaving a considerably smaller pool of breeding candidates and thus potentially creating a genetic bottleneck. Indeed, with identification of an ever-increasing number of mutations that are either the direct cause of, or are influential on the development of, disease, breeders are being faced with an increasing number of objective traits relating to diseases. Therefore, the focus of future breeding strategies will have to be on compensatory pairings of candidates, since very few individuals will have a clean bill of health for all objectives.

