# Supplementary material for: Letter to the editor regarding an autosomal recessive mutation in SCL24A4 causing enamel hypoplasia in Samoyed and its relationship to breed-wide genetic diversity
Source: Canine Genet Epidemiol. 2018 May 1;5:4. doi: 10.1186/s40575-018-0059-7 (PMC5930769; doi:10.1186/s40575-018-0059-7)
Supplement: Related texts

# Reply: Authors’ response

**Authors:** Niels C. Pedersen, Bonnie Shope, Hongwei Liu

Professors Nicholas and Mellersh and Dr. Lewis were concerned about the methodology that led us to conclude that Samoyed lack genetic diversity. They then used these concerns to frame a personal opinion meant to be reassuring to breeders. In doing so, they ignored the major theme of our study, which was to describe a heretofore unrecognized autosomal recessive disorder in Samoyed and the historic and genetic context in which it arose. As veterinarians, we were deeply concerned about identifying health problems of a genetic nature and providing tests to deal with them. We also wanted to make the study a teaching as well as research experience for all breeders.

Their first criticism involved the use of 33 microsatellite markers across only 25/38 autosomes. This was linked to another question pertaining to why microsatellites were even being used at all, given modern SNP technology. The microsatellites chosen for this study were vigorously selected for their ability to discern genetic differences by the International Society for Animal Genetics, with addition of several new markers from our Veterinary Genetics Laboratory. The microsatellites are known to be highly polymorphic and have been used successfully for many studies concerning parentage and genetic diversity. Some of the added markers were for the DLA class I and II regions, which contains genes concerned with immune responses. This region is in strong linkage disequilibrium, with one copy inherited by descent from each parent. It is extremely difficult to define the DLA region using SNPs due to the numerous polymorphisms, but it is very efficient and accurate to track this region using microsatellites. DLA haplotypes are powerful identifiers of breed founders. We were also concerned about the fact that our markers did not cover every chromosome and added 25 more microsatellites to cover missing chromosomes. We found that the additional microsatellites, although carefully selected, did not add anything to the power of the current 33 markers to study breed-wide genetic diversity and relatedness. We also compared genetic diversity in Standard Poodles by both microsatellites and 170K SNPs and found no major differences. This same microsatellite panel has been used to successfully study genetic diversity in a number of dog breeds and this information can be found on the Veterinary Genetics Laboratory website - https://www.vgl.ucdavis.edu/services/dog.php. We are confident, therefore, that the genetic diversity we were measuring in Samoyed was accurate as described and that employing large panels of SNPs would not significantly alter the conclusions.

The numbers, relatedness, and geographic distribution of Samoyed was questioned, with the implication that we underestimated the genetic diversity in Samoyed compared to earlier pedigree and SNP studies. Although we failed to mention it, more than one hundred of the DNA samples were from Samoyed from around the world submitted to the VGL for other genetic testing and not for the ARAI mutation. One hundred eighty-four Samoyed from around the world is also not a small sampling, at least compared to virtually all other breed studies one might find in the literature. The fact that the 184 Samoyed were genetically similar across the world adds also indicates that most of the existing genetic diversity in the breed was represented in this population.

The reviewers cited other studies involving pedigrees and SNPs as evidence that we may have under-estimated genetic diversity in Samoyed. They failed to mention that the conclusions of these studies were the same. The pedigree study of Bell concluded that the breed had a narrow base of founders and that deep pedigrees showed extensive sharing of the same individuals. A pedigree study conducted by the UK Kennel Club estimated that the effective population size for UK Samoyed was 64.7 dogs. Both studies agreed with our conclusion that genetic diversity in the breed was not great, although adequate. The SNP study quoted by Sorsa used the MyDogDNA array containing around 6788 SNPs, but the study only involved 50 Samoyed and the author admitted that the number was too small to make any conclusion other than that diversity appeared adequate. We also concluded that genetic diversity in Samoyed was derived from a small founder population, but that it was adequate. We have limited faith in pedigrees as sole determinants of genetic diversity.

The second half of the comments pertained to what should be done when a new genetic disorder is identified in a breed and a test for that mutation provided. We did offer three alternative solutions to breeders regarding autosomal recessive enamel hypoplasia: 1) ignore it, 2) eliminate it, or 3) breed around it. We believe that the professors and Dr. Lewis were saying the same things, although they offer a fourth option, i.e., to quickly reduce the incidence of the mutation by testing to an “acceptable” level and live with it. We described the reasons why each of our three approaches might be selected. If the defect is at very low incidence, the genetic diversity sufficient, and breeders are good about random mate selection, ignoring the mutation is a viable option. The chances of getting affected dogs would be extremely small. However, if there is adequate genetic diversity and the incidence of the defect is low, it might be a good idea to eliminate the trait on the perchance that it might be amplified later by some sort of artificial genetic selection (e.g., popular sire effect). We provided our best possible scientific evidence that eliminating the mutation in Samoyed could be accomplished without loss of genetic diversity. We do agree with the reviewers that eliminating genetic traits can lead to serious inbreeding in situations where the incidence of the mutation is high and breed-wide genetic diversity low. Such a situation arose for pug dog encephalitis. We identified a region in the DLA that identified carriers of the trait, but when we looked at the incidence of the causative region, over one-third of individuals in the breed were carriers and genetic diversity for the breed was low. Eliminating carriers would seriously reduce existing genetic diversity. Therefore, a recommendation was given to select around the trait by careful mate selection. Fortunately, the trait was recessive, so breeders could select for Normal/Normal or Normal/Carrier puppies. We stand by our conclusion that the ARAI mutation can be safely eliminated from the breed.

We agree that the reviewer’s description of the problems that breeders face when having to select against multiple simple and complex genetic traits when given a small number of available dogs, low genetic diversity and geographic limitations. However, the professor’s and Dr. Lewis’ major conclusion that “the focus of future breeding strategies will have to be on compensatory pairings of candidates, since very few individuals will have a clean bill of health for all objectives” is a bit defeatist. It is also dismissive of the research that went into our study and an admission that all breeds are in trouble and the best that can be done is to choose the least objectionable pairings. Since this is precisely what good breeders are already doing, their suggestion is another reassurance from certain geneticists to breeders that “all is right, and nothing needs to change.” We have hopefully shown Samoyed breeders that they have not yet reached a point of great concern but forewarned them that Samoyed around the world come from a limited genetic foundation that needs to be carefully managed. The emergence of ARAI in the breed points to the problems facing many other breeds with small numbers and limited genetic diversity. The fact that Samoyed breeders have retained reasonable health through their breed’s existence is a plus.
